# The association between population drinking and ischemic heart disease mortality in educational groups

**DOI:** 10.1093/alcalc/agad033

**Published:** 2023-05-18

**Authors:** Thor Norström, Jonas Landberg

**Affiliations:** Swedish Institute for Social Research (SOFI), Stockholm University, Stockholm 109 91, Sweden; Department of Public Health Sciences, Stockholm University, Stockholm 106 91, Sweden; Department of Clinical Neuroscience, Karolinska Institutet, Stockholm 171 77, Sweden

**Keywords:** alcohol, heart disease mortality, time-series, Sweden, education

## Abstract

A large number of observational studies have found a J-shaped relationship between alcohol intake and ischemic heart disease (IHD) risk. However, some studies suggest that the alleged cardio-protective effect may be an artifact in the way that the elevated risk for abstainers is due to self-selection on risk factors for IHD. The aim of this paper is to estimate the association between alcohol and IHD-mortality on the basis of aggregate time-series data, where the problem with selection effects is not present. In addition, we will analyze SES-specific mortality to investigate whether there is any socio-economic gradient in the relationship at issue. SES was measured by educational level. We used IHD-mortality in three educational groups as outcome. Per capita alcohol consumption was proxied by Systembolaget’s alcohol sales (litres of alcohol 100% per capita 15+). Swedish quarterly data on mortality and alcohol consumption spanned the period 1991Q1–2020Q4. We applied SARIMA time-series analysis. Survey data were used to construct an indicator of heavy SES-specific episodic drinking. The estimated association between per capita consumption and IHD-mortality was positive and statistically significant in the two groups with primary and secondary education, but not in the group with postsecondary education. The association was significantly stronger the lower the educational group. Although the associations were generally stronger for males than for females, these differences were not statistically significant (*P* > 0.05). Our findings suggest that the detrimental impact of per capita consumption on IHD-mortality was stronger the lower the educational group.

## Teaser

**Short Summary:** Using Swedish quarterly data on alcohol consumption and IHD-mortality spanning the period 1991–2020 we found that increased per capita consumption was associated with statistically significant increases in IHD-mortality in the two lowest educated groups (those with primary and those with secondary education), while the association was statistically insignificant in the group with postsecondary education.

## Introduction

Although most of the research on the association between alcohol consumption and ischemic heart disease (IHD) mortality is based on observational individual-level data, there are some studies (e.g. Hemström, 2001; Ramstedt, 2006; Ramstedt, 2009) that rely on aggregate data to address the topic at the population level. This study expands the focus by estimating the association between population drinking and IHD-mortality across various socio-economic groups.

Alcohol consumption has been found to be a significant risk factor for a large number of fatal outcomes, including violent deaths, cirrhosis of the liver, and various forms of cancer (Rehm *et al*., 2017). However, the relationship between alcohol use and the risk of IHD mortality is contested (see e.g. Andréasson, 1998; Shaper, 1990). Numerous observational studies have thus found a J-shaped relationship between alcohol intake and IHD-risk, implying that an elevated risk is observed for abstainers and heavy drinkers, while there would be a cardio-protective effect of moderate consumption (see Corrao *et al*., 2000; Hemström, 2001; Ronksley *et al*., 2011; Day and Rudd, 2019; Hoek *et al*., 2022 for reviews). A number of possible biochemical mechanisms underlying the alleged cardioprotective effect of alcohol have been suggested. These include increased HDL cholesterol, reduced clotting activity of the blood, and healthier fibrinogen concentrations, but none of these mechanisms has as yet been firmly established (Agarwal, 2002). However, some studies suggest that the alleged cardio-protective effect may be spurious, or at least biased upward. For instance, due to self-selection the group of non-drinkers is a selective category which may contain individuals who abstain from alcohol because of health issues or previous alcohol problems (Shaper and Wannamethee, 2000; Roerecke and Rehm, 2010b; Zhao *et al*., 2017). Moreover, the increased IHD-risk of abstainers may be due to lifestyle confounding, since this group generally has a less favorable life-style than light or moderate drinkers, with e.g. elevated levels of unhealthy diets and low physical activity (Fekjaer, 2013). Further, reviews of observational studies suggest that the association between alcohol use and IHD-risk may be modified by drinking patterns, so that moderate consumption in combination with a detrimental drinking pattern characterized by heavy episodic drinking (HED) does not seem to confer any cardioprotective effect (Roerecke and Rehm, 2010a, 2014).

Another approach for assessing the link between alcohol and cardiac risk is to analyze aggregate-level data, e.g. using time-series data to estimate the impact of changes in per capita consumption on IHD-mortality. This approach has the potential of elucidating a question that is highly relevant from a public health perspective: provided that moderate alcohol consumption has a cardioprotective effect, while heavy consumption has a deleterious impact; what is the potential net-effect of an increase in total consumption? Further, a methodological advantage of this approach is that the problem of selection effects is avoided (Norström and Skog, 2001). To see this, consider the following model pertaining to individual-level data:


(1) \begin{equation*} {\mathrm{Y}}_{it}={\mathrm{C}}_i+\mathrm{b}\cdot{\mathrm{X}}_{it}+{\mathrm{e}}_{it} \end{equation*}

where Y*_it_* is the response of individual *i* at time *t*; C*_i_* represents unmeasured time-invariant factors that are correlated with the outcome; and e*_it_* is a random error term. The potential bias in the estimation of b is thus due to the possible correlation between C*_i_* and X*_it_* that may arise because of self-selection. Now consider the model in aggregate form:


(2) \begin{equation*} {\mathrm{Y}}_t=\mathrm{C}+\mathrm{b}\cdot{\mathrm{X}}_t+{\mathrm{e}}_t \end{equation*}

As we can see the aggregation turns C*_i_* into a constant which by definition is uncorrelated with X*_t_*, and thus this source of bias is eliminated. To illustrate, let Y indicate IHD-mortality, C health status, and X alcohol consumption. If poor health status in addition to increasing the individual’s IHD-risk also has a reducing effect on the individual’s alcohol consumption, the ensuing correlation between C and X will produce a downward bias in the estimate of b in Eq. 1. If we assume that the population prevalence of poor health status does not affect alcohol consumption per capita, the estimate of b in Eq. 2 will not be plagued by the bias due to self-selection. If this assumption is invalid (i.e. the prevalence of poor health does affect per capita alcohol consumption), the estimate of b in Eq. 2 will be plagued by omitted variable bias.

The study by Hemström (Hemström, 2001) is one of the first investigations of the aggregate association between IHD-mortality and per capita alcohol consumption. On the basis of time-series data for 15 western European countries spanning the period 1950–1995, the results showed statistically insignificant negative and positive effect estimates of per capita alcohol consumption on IHD-mortality. In a study based on Canadian time-series data covering the period 1950–1998, Ramstedt (Ramstedt, 2006) found a weak, but significant, positive effect of changes in per capita alcohol consumption on IHD-mortality; that is, increased consumption was associated with increased mortality. Using panel data for the US states from 1950 to 2002, Kerr et al. (Kerr *et al*., 2011) reported an overall, significantly positive effect of per capita consumption on IHD-mortality. Per capita beer consumption appeared to have a protective effect, which, however, was outweighed by the deleterious impact of spirits consumption. The authors interpreted the protective effect of beer as a result of the low to moderate intake patterns associated with protective effects being more common among beer drinkers.

The importance of drinking patterns is echoed also in population-level studies. Thus, a multi-level analysis of aggregate data comprising 74 countries (Gmel *et al*., 2003) suggested that the impact of per capita alcohol consumption on heart disease mortality was contingent on the societal pattern of drinking: in countries with regular moderate drinking with meals (e.g. France, Italy, Spain), per capita consumption had a cardioprotective effect, while in countries where drinking tends to be characterized by irregular and heavy drinking (e.g. Russia) increased per capita consumption was associated with increased IHD-mortality. Additional evidence pertaining to Russia is reported by (Ramstedt, 2009); on the basis of an ARIMA-analysis of data spanning the period 1959–1998 a positive and statistically significant relationship was found between per capita consumption and male IHD-mortality. The analysis was restricted to men, because binge drinking (the pattern of drinking hypothesized to be associated with an elevated risk of IHD) is predominantly a male phenomenon in Russia.

### This study

Previous population-level studies have used IHD-mortality for the entire population as outcome (sometimes stratified by gender or age). This study aims to broaden the focus by performing SES-specific analyses; more precisely we will use Swedish quarterly time-series data to estimate the relationship between per capita alcohol consumption and IHD-mortality in three educational groups. Another aim is to elucidate the impact of drinking patterns on the relationship at issue. This will be accomplished by utilizing SES-specific data on HED. Considering that disadvantaged SES-groups tend to have elevated levels of HED in Sweden (Landberg *et al*., 2020, 2021)), an obvious hypothesis is that the aggregate alcohol and IHD association will differ across SES groups, with more detrimental alcohol effects in lower SES groups.

### Data

#### Socio-economic status

We used education as indicator of socio-economic status. Education, together with occupation and income, is one of the main dimensions for classifying SES and is often used in studies of SES-gradients in mortality (Miech *et al*., 2011). Education has the advantage of being more stable over time and is less likely to be influenced by reversed causation than occupation and income, i.e. that a person’s occupation and/or income may be adversely affected by his/hers misuse of alcohol. We used three educational groups: (i): primary education (no qualifications, primary years of schooling, 9 years or less); (ii): secondary education (upper secondary school education, 10–12 years); and (iii): postsecondary education (college or university education, 13+ years). Data source: Swedish Register of Education.

#### Outcome

IHD mortality (ICD-9: 410-414; ICD-10: I20-I25). Data source: National Board of Health and Welfare.

#### Control variable

It seems warranted to control for smoking, as it may be associated with IHD-mortality as well as per capita alcohol consumption. In the absence of quarterly smoking data we used lung cancer mortality (ICD-9: 162; ICD-10: C33-C34) as proxy. The feasibility of this approach is supported by (Bilal *et al*., 2014). Data source: National Board of Health and Welfare.

Information on educational level was linked from the Swedish Register of Education to the mortality data through personal identification numbers. We constructed age-standardized mortality rates per 100 000 in the age-group 25–79 years for the three educational groups. (Both the outcome (IHD-mortality) and the control series (lung cancer mortality) were SES-specific.) We chose the lower age limit (25) because the highest level of education has normally been attained at that age. The upper age limit was motivated by the finding that the accuracy of the cause-of-death classification is poorer in higher ages (Meslé, 2007; Alpérovitch *et al*., 2009; Mieno *et al*., 2016).

*Per capita alcohol consumption* was proxied by Systembolaget’s sales expressed as litres of alcohol (100%) per inhabitant aged 15 years and above.

All time-series data are quarterly; the mortality data span the period 1991Q1–2020Q4, and the alcohol series covers the period 1989Q1–2020Q4.

#### Drinking patterns

To depict SES-specific drinking patterns we used individual-level data from a database collected within the Monitoring Project: an ongoing monthly telephone survey including questions about self-reported drinking habits. A nationally representative sample of the Swedish general population aged 16–80 years is randomly drawn on a monthly basis. Interviews are then conducted until 1500 respondents have been interviewed each month, resulting in a repeated cross-sectional sample of ~18 000 respondents per year. The monthly non-response rate ranges between 40 and 60%, and tends to increase over time (Trolldal and Leifman, 2015). The analytical sample for this study included the years 2004 to 2020 and respondents aged 25–79 years, without missing information on education or alcohol use, amounting to 92 624 females and 88 358 males after excluding non-drinkers.

The indicator of drinking patterns (henceforth, drinking pattern score, DPS for short) was constructed as the percentage of all drinks (consumed during the past 30 days) consumed during heavy episodic drink occasions (HED occasions), that is:


(3) \begin{align*} &DPS=\nonumber\\ & = 100\ast\frac{Number\, of \, drinks\, consumed\ in\, HED\, occasions\, during\, the\, past\, 30\, days}{Number\ of\ drinks\ consumed\ during\ the\ past\ 30\ days} \end{align*}

The numerator was based on a question on how often during the past 30 days the respondent on one occasion had consumed alcohol equivalent to at least six standard glasses (exemplified as a whole bottle of wine, four cans (50 cl) of strong beer (5,5% ABV), six cans of medium strength beer (3.5% ABV), or 25 cl spirits). The nine response alternatives ranged from “Never” (coded 0) to “Practically everyday” (coded 27.5). The response to this question was multiplied by 6 (the threshold we used for HED), which yields the number of drinks consumed in HED occasions during the past 30 days. The denominator was calculated on the basis of a beverage-specific quantity and frequency scale. This scale combines questions on how often spirits, wine, beer, and cider have been consumed during the past 30 days, with the typical amount consumed per occasion. The answers were then summarized into a measure of overall drinking during the past 30 days, expressed in litres of 100% alcohol. This was then transformed into number of standard glasses by multiplying with 65.75 (1 standard glass = 12 g alcohol. 1 litre 100% alcohol = 789 g = 65.75 standard glasses (789/12)).

### Statistical analysis

The relationship between per capita consumption and IHD-mortality was analyzed by applying the technique of SARIMA-modelling (seasonal autoregressive integrated moving average model) (Box *et al*., 2008). Non-stationarity in the form of time trends was removed by regular or seasonal differencing. The noise (error) term, which includes explanatory variables not considered in the model, is allowed to have a temporal structure that is modelled and estimated in terms of regular and seasonal autoregressive or moving average parameters. A SARIMA-model is specified as: (p, d, q) (P, D, Q, M), where the first bracket represents the model’s non-seasonal (regular) part, and the second bracket specifies the seasonal part. The order of the autoregressive parameter in the model's non-seasonal part is indicated by p, while d indicates the order of regular differencing, and q is the order of the moving-average parameter. The symbols in the second bracket have the corresponding seasonal significance, while M is the number of periods per season. The model residuals should not differ from white noise; this was tested using the Box–Ljung Q statistics (Ljung and Box, 1978).

The plausible time-lag in the relationship between per capita alcohol consumption and IHD-mortality in the context of quarterly data was considered using a weighted alcohol variable of past and present observations with geometrically declining lag-weights, an approach that is often applied in this kind of analyses (Norström and Skog, 2001). The weighted alcohol variable (*AW*) was constructed as follows:


(4) \begin{align*} {AW}_t =&\ {A}_t+\mathrm{\lambda} \ast{\mathrm{A}}_{t-1}+{\mathrm{\lambda}}^2\ast{A}_{t-2}+{\mathrm{\lambda}}^3\ast{A}_{t-3}+{\mathrm{\lambda}}^4\ast{A}_{t-4}\nonumber\\&+{\mathrm{\lambda}}^5\ast{A}_{t-5}+\dots{\mathrm{\lambda}}^n\ast{A}_{t-n} \end{align*}

There was a truncation at lag 15 (i.e. *n* = 15); further, the lag weights were rescaled to sum to unity. The higher the value of the lag parameter (λ), the slower the mortality response, i.e. the higher the impact of past observations. The lag parameter was fixed at 0.99. Exploratory analyses indicated that decreasing the value of λ did not improve the model fit.

Possible effects of the introduction of ICD-10 (1997) were captured by a dummy variable, taking the value 0 prior to 1997Q1, and 1 otherwise.

Using Monitor data, we applied ordinary least squares regression (Stata command Regress) to estimate adjusted SES-specific means of drinking patterns, i.e. the mean value of the DPS in each educational group, stratified by gender. The means were adjusted for the effects of region and age. Age was included as a continuous variable, while region was a categorical variable with three values: 1 = northern Sweden, 2 = mid-Sweden, and 3 = southern Sweden (see (Trolldal *et al*., 2017) for exact delineations). The adjusted means were obtained through the post-estimation procedure Margins in Stata V.17.

All statistical analyses were performed with Stata V.17 (StataCorp, College Station, TX, USA).

### Ethical considerations

The study was approved by the regional ethics committee in Stockholm (Dnr 2018/2018-31/5).

**Figure 1:**
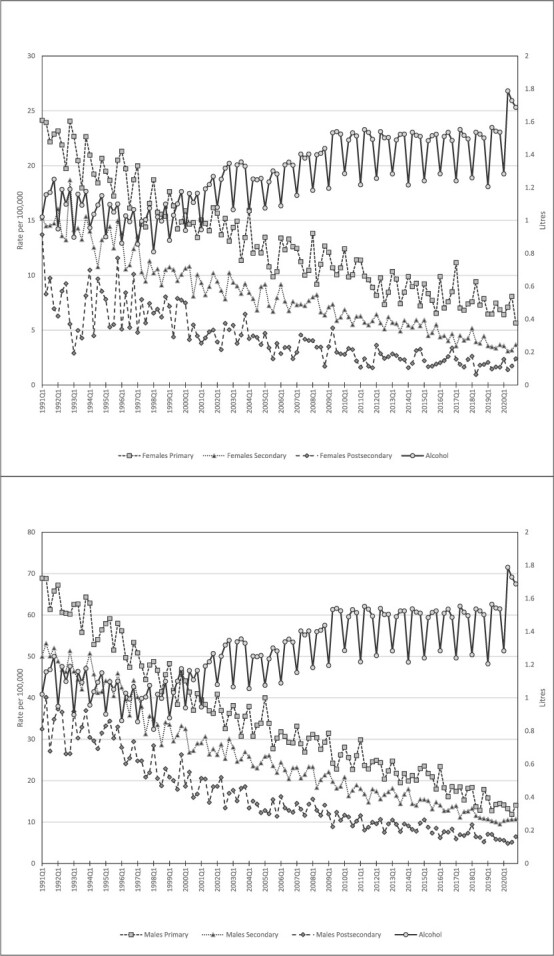
Trends in IHD-mortality in three educational groups and per capita alcohol consumption. Quarterly data 1991Q1–2020Q4. Females (top); Males (bottom).

## Results

Figure 1 depicts a marked decline in IHD-mortality which is a continuation of a long-term decrease that “has been fueled by rapid progress in both prevention and treatment, including precipitous declines in cigarette smoking, improvements in hypertension treatment and control, widespread use of statins to lower circulating cholesterol levels, and the development and timely use of thrombolysis and stents in acute coronary syndrome to limit or prevent infarction” (Mensah *et al*., 2017). The SES-gradient in mortality was stronger for females than for males; the ratio between the group with primary and the group with postsecondary education was 3.014 for females, and 2.106 for males (average for the whole period).

The estimates from the SARIMA-modelling (Table 1) suggest statistically significant positive effects of per capita consumption on IHD-mortality in the two groups with primary and secondary education, whereas the estimate in the group with postsecondary education was statistically insignificant. This pattern was observed for females as well as males. The estimates express the change in the mortality rate associated with a 1-litre increase in per capita consumption. For instance, a 1-litre increase in per capita consumption (on a quarterly basis) would give rise to 13.181 additional deaths (on a quarterly basis) per 100 000 in males with primary education, while the corresponding number for males with secondary and postsecondary education was 8.275 and − 3.627, respectively (1 litre 100% alcohol corresponds to 65.75 standard glasses à 12 g). That is, the estimated effect of an increase in alcohol consumption was weaker the higher the educational group; this gradient was statistically significant for females as well as males. Although the estimated alcohol effects were generally stronger for males than for females, these differences were not statistically significant (*P* > 0.05). Further, none of the estimated effects of lung cancer mortality were statistically significant. The residuals from all estimated models were white noise.

**Table 1:** Estimated effects of per capita alcohol consumption on IHD-mortality. Based on SARIMA modelling of quarterly data 1991Q1–2020Q4

			Alcohol			Lung cancer						
	Education	DPS	EST	SE	*P*	EST	SE	*P*	Q	p(Q)	Model	
Males	Primary	21.456	13.181	6.497	0.042	0.292	0.226	0.197	4.482	0.214	(2,0,0)(0,1,1,4)	
	Secondary	18.039	8.275	3.884	0.033	0.096	0.189	0.611	1.689	0.793	(2,0,0)(0,1,1,4)	
	Postsecondary	15.605	−3.627	3.516	0.302	0.046	0.192	0.811	1.076	0.898	(0,0,0)(0,1,1,4)	
F-test for heterogeneity (p)		107.68 (<0.001)	4.463		(0.005)							
Females	Primary	13.498	4.346	1.626	0.008	−0.095	0.131	0.470	3.195	0.074	(0,0,0)(0,1,1,4)	
	Secondary	12.217	3.509	1.119	0.002	−0.216	0.118	0.066	1.788	0.181	(0,0,0)(0,1,1,4)	
	Postsecondary	8.571	−0.728	1.982	0.713	−0.102	0.117	0.385	0.067	0.796	(0,0,0)(0,1,1,4)	
F-test for heterogeneity (p)		57.12 (<0.001)	3.828		(0.012)							

DPS: drinking pattern score

Q: Box–Ljung test for residual correlation (lag 4)

**Figure 2:**
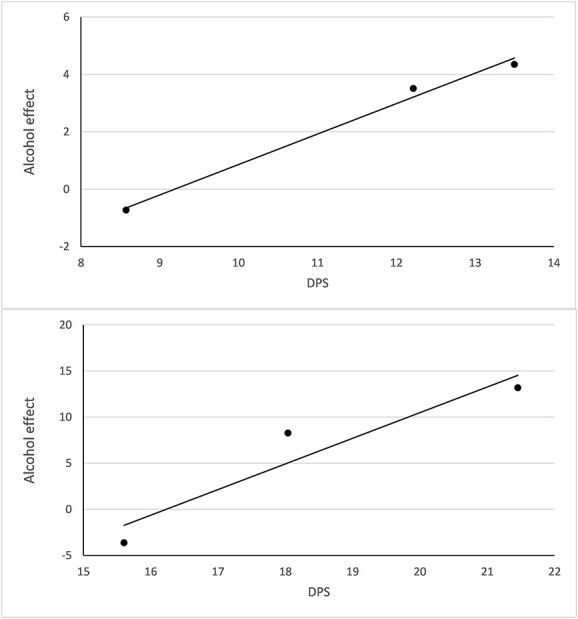
Estimated effect of per capita alcohol consumption on IHD-mortality (Table 1, column 4) plotted against DPS (Table 1, column 3). Females (top); Males (bottom).

We also observed marked educational differences in our indicator of drinking patterns: the lower the educational group, the more harmful the drinking pattern. There was a fairly close association between the DPS and the estimated alcohol effect: that is, the higher the DPS (i.e. the more detrimental the drinking pattern), the stronger the estimated alcohol effect (Fig. 2).

The dummy variable capturing the possible impact of the introduction of ICD-10 was negative (i.e. associated with a lowered mortality rate), and statistically significant (*P* < 0.05) in all estimated models except for the model pertaining to females with postsecondary education (*P* = 0.114).

## Discussion

The number of individual-level studies addressing the association between alcohol consumption and IHD-mortality is abundant, whereas population level studies on this subject are scarce. This study broadens the focus of the latter approach; on the basis of Swedish quarterly data spanning the period 1991–2020 we estimated the relationship between per capita alcohol consumption and SES-specific IHD-mortality, more specifically, IHD-mortality in three educational groups. Consistent with most other population level studies on this subject, for a review, see (Norström and Ramstedt, 2005), our findings did not suggest any general cardio-protective effect of alcohol, although our findings do not preclude the existence of a cardio-protective effect in some specific segments of the population, e.g. people who drink at a low and stable level, and score low on cardiac risk factors. However, we found that increased per capita consumption was associated with statistically significant increases in mortality in the two lowest educated groups (those with primary and those with secondary education), while the association was statistically insignificant in the group with postsecondary education. The gradient in estimated alcohol effects was statistically significant. This differential effect of alcohol use on IHD risk across educational groups is partly in line with results of a recent individual-level cohort-study. Based on register data from three population-based cardiovascular health surveys in Norway (*n* = 207 394), Degerud et al. (Degerud *et al*., 2018) found that very frequent consumption was associated with increased risk of CVD mortality, but only among those with low socioeconomic position. The phenomenon has also been observed at the population level in a time-series analysis of Swedish data, showing that the association between per capita consumption and alcohol-related mortality was markedly stronger the lower the educational group (Norström and Landberg, 2020).

One possible explanation of our findings is that the temporal variations in population drinking are not synchronized across SES-groups so that increases in total consumption have been disproportionally allocated to low-SES groups. According to Skog, such deviations from the rule of the collectivity of drinking (Skog, 1985) can be expected if social barriers hamper the synchronizing contagion process across social strata, or if external factors, such as buying power, develop differently across SES-groups (Norström and Romelsjö, 1998). However, this notion is not supported by the findings pertaining to Sweden reported by Landberg et al. (Landberg *et al*., 2021). They found that annual changes in overall mean consumption during the study period (2004–2014) were reflected in collective shifts in drinking across educational groups. That is, the annual changes in mean volume of consumption in groups with primary, secondary, and postsecondary education were in the same direction and of a similar magnitude during the study period. Our findings thus resemble results from individual level research focusing on The Alcohol Harm Paradox (Katikireddi *et al*., 2017; Probst *et al*., 2020), implying that a given volume of alcohol yields more harm in low-SES groups than in high-SES groups.

SES-differences in drinking patterns has been proposed as one possible explanation to The Alcohol Harm Paradox (Boyd *et al*., 2022). Our findings are indeed consistent with this notion; we found a significant SES-gradient in the DPS, implying a more harmful drinking pattern the lower the educational group. Further, there was a fairly close match between the DPS and the estimated alcohol effect in the expected direction. Finally, it is possible that our results also reflect that other behavioral or social risk-factors (e.g. obesity, high cholesterol levels, and poor working conditions) cluster in low-SES groups and may interact with alcohol use, resulting in an elevated IHD-risk.

Our findings have important policy implications; given that changes in per capita alcohol consumption appear to have a larger impact on IHD-mortality among more disadvantaged SES-groups, policy measures that effectively regulate the total consumption in society will have the potential of reducing the social gradient of this outcome.

### Strengths and limitations

This is the first study that addresses the relationship between population drinking and SES-specific IHD-mortality. By integrating individual-level and population-level data we could explore the importance of drinking patterns, more specifically HED, for the variations in the magnitude of the SES-specific relationships at issue. However, our findings are specific to Sweden and cannot be generalized to countries with other alcohol cultures and drinking patterns. Another limitation is that we used Systembolaget’s sales as a measure of alcohol consumption and thereby exclude unrecorded alcohol consumption from our analyses. Still, a recent study focusing on SES-differences in unrecorded consumption in Sweden during the study period found no significant differences between educational groups in consumption of unrecorded alcohol as a whole (Norström *et al*., 2021). Consequently, while not including unrecorded alcohol consumption may have biased all our estimated alcohol effects upwards (in case of a positive association between recorded and unrecorded alcohol consumption) or downwards (in case the association between recorded and unrecorded consumption is negative), it should not affect our estimated social gradient in the alcohol effect on IHD mortality.

## Conclusions

Based on Swedish quarterly data for the period 1991–2020, we found a differential association between per capita consumption and IHD-mortality, with stronger and more harmful alcohol effects the lower the educational group. Our findings thus add support to the notion that the Alcohol Harm Paradox may be found also at the population level.

## Author contributions

Thor Norström (Conceptualization-Equal, Data curation-Equal, Formal analysis-Equal, Funding acquisition-Equal, Investigation-Equal, Methodology-Equal, Project administration-Equal, Resources-Equal, Software-Equal, Supervision-Equal, Validation-Equal, Visualization-Equal, Writing – original draft-Equal, Writing – review & editing-Equal), Jonas Landberg (Conceptualization-Equal, Data curation-Equal, Formal analysis-Supporting, Funding acquisition-Equal, Investigation-Equal, Methodology-Equal, Project administration-Equal).

*Conflict of interest*. None declared.

## Funding

This study was funded by the Swedish Research Council for Health, Working life and Welfare (Forte) [grant number 2017-01769] and Systembolaget’s Research Council (Systembolagets Alkoholforskningsråd) [grant number FO2017-0084].

## Data availability

Request for Swedish mortality data should be approved by the Swedish Ethical Review Authority (https://etikprovningsmyndigheten.se/en/).
